# Nutrient dynamics and GHG emissions in *Azolla* and *Typha* based cultivation on inundated former agricultural soils

**DOI:** 10.1007/s11104-025-08032-y

**Published:** 2025-11-17

**Authors:** Renske J. E. Vroom, Alfons J. P. Smolders, Leon P. M. Lamers, Bas P. van de Riet, Sarian Kosten

**Affiliations:** 1 Department of Ecology, Radboud Institute for Biological and Environmental Sciences, Radboud University, Heyendaalseweg 135, 6525 AJ Nijmegen, The Netherlands; 2 Institute of Botany and Landscape Ecology, University of Greifswald, partner in the Greifswald Mire Centre, Soldmannstraße 15, 17489 Greifswald, Germany; 3 B-WARE Research Centre, Toernooiveld 1, Nijmegen, 6525 ED The Netherlands

**Keywords:** Phosphate, Methane, Wetland, *Azolla filiculoides*, Mesocosm, Rehabilitation

## Abstract

**Background and aims:**

Restoration and novel creation of wetlands is crucial as they store and purify water, sequester carbon, and are biodiversity hotspots. However, wetland rehabilitation on agriculturally-used soils typically causes water quality issues, low biodiversity and high methane emissions. To tackle these challenges in a novel, cost-effective way, *Azolla filiculoides*, a water-fern capable of nitrogen fixation and phosphorus (P) accumulation, could be cultivated after inundation to simultaneously extract nutrients and provide a commercial product.

**Methods:**

We cultivated *A. filiculoides* and a polyculture of *A. filiculoides* and *Typha angustifolia*, an emergent macrophyte, on two P-rich former agricultural mineral soils in an outdoor mesocosm experiment during two years. We measured nutrient dynamics in soil, water, and biomass, diffusive and ebullitive methane (CH_4_) emissions, and nitrous oxide emissions.

**Results:**

Open water controls showed substantial P mobilisation to the surface water and were dominated by microalgae or emergent macrophytes. *Azolla* cultivation lowered surface water P concentrations, but did not negate them in the most P rich soil. Infestation with the Azolla weevil (*Stenopelmus rufinasus*) severely constrained *Azolla* growth. Thus, P extraction rates were moderate: up to 38 kg ha^−1^ yr^−1^ in the *Azolla* monoculture, and 67 kg ha^−1^ yr^−1^ in the polyculture with *T. angustifolia*. Methane emissions were substantial and ebullition-dominated in all treatments, and not affected by *Azolla* cultivation.

**Conclusion:**

*Azolla* cultivation shows potential in the transition from agriculture to wet nature, while recovering P from former agricultural soils. Remaining challenges include pest control, product development, and technologies for large-scale implementation.

**Supplementary Information:**

The online version contains supplementary material available at 10.1007/s11104-025-08032-y.

## Introduction

Wetland ecosystems play an essential role in the landscape: they store and purify water, sequester carbon (C), and harbour a large abundance of organisms (Zedler and Kercher 2005). However, wetlands are declining worldwide at alarming rates due to drainage, farmland conversion, pollution, and other human impacts (Davidson et al. 2018; Fluet-Chouinard et al. 2023). Wetland degradation leads to increased flood and fire risk, water quality issues, intense C emissions, and severe biodiversity losses (Albert et al. 2021; Kingsford et al. 2016; Mitsch et al. 2013). Wetland conservation, restoration, and novel creation is therefore an urgent challenge, even more so in light of global change (Erwin 2009).

Wetlands that have been converted to arable land frequently have a history of intense fertilization. As a result, their soils contain substantial nutrient legacies, particularly of nitrogen (N) and phosphorus (P) (Lamers et al. 2006; Macdonald et al. 2012; Smolders et al. 2006). Rewetting or inundation, a first step in wetland restoration or creation, can lead to excessive nutrient mobilization, particularly of ammonium (NH_4_^+^) and phosphate (PO_4_^3−^) (Pant and Reddy 2003; Zak and Gelbrecht 2007). High nutrient loads hamper rehabilitation of wetland functions, resulting in poor water quality, limited C sink recovery (e.g. due to low primary production and high rates of decomposition), and low biodiversity (Antonijević et al. 2023; Kreyling et al. 2021).

To improve or rehabilitate wetland functions on agriculturally used soils, the temporary cultivation of fast-growing wetland plants provides opportunities (Gaudig et al. 2018; Rezania et al. 2021; Vymazal 2007; Wichtmann et al. 2016). Wetland plants take up nutrients, which can be removed by harvesting. Meanwhile, their biomass has various uses, depending on the cultivated species (Abel and Kallweit 2022). One such fast-growing species is the free-floating waterfern *Azolla filiculoides* (Wagner 1997). Due to its symbiosis with nitrogen-fixing cyanobacteria *Nostoc*/*Anabaena azollae* (Peters and Meeks 1989), *Azolla* shows very high clonal growth rates, even in N-limited conditions (Wagner 1997). *Azolla* can hyperaccumulate N, P, and heavy metals, and is therefore often used as a biofertiliser in rice systems and in phytoremediation of polluted water (Hendriks et al. 2023; Kimani et al. 2020; Sood et al. 2012). Because of these properties, it is an excellent candidate for P extraction from former agricultural lands that are converted to wetlands (Temmink et al. 2018; Vroom et al. 2024). Meanwhile, its protein-rich biomass offers various economically viable uses, for instance as animal feed or biofertiliser, depending on its composition (Brouwer et al. 2018).

As P availability is an important limiting factor for *Azolla* growth (Temmink et al. 2018), *Azolla* cultivation requires high soil PO_4_^3-^ mobilisation rates to the surface water. When *Azolla* can grow unbridled, the floating mat can limit O_2_ diffusion into the surface water, resulting in anoxia (Pinero-Rodríguez et al. 2021; Sheppard et al. 2006). In turn, anoxia can enhance the release of P from the soil to the water layer, potentially accelerating soil P removal by *Azolla*. However, *Azolla* may not reduce surface water O_2_ concentrations when harvested at least every month, despite completely covering the water surface in-between harvests (Vroom et al. 2024). Therefore, *Azolla* only thrives when sediment P mobilisation is sufficiently high in oxic conditions, i.e. when porewater iron (Fe) to P ratios are low (< 10), and porewater P concentrations are high (Geurts et al. 2010, 2008; Tang et al. 2016; Vroom et al. 2024). When these conditions were met, high P extraction rates could be realised in a greenhouse experiment (Vroom et al. 2024). Under field conditions, however, P extraction rates may be lower due to seasonally lower temperatures and other external factors (e.g. diseases or feeding by insects). On the other hand, an increase in decaying organic matter, e.g. through shed *Azolla* roots, may lead to surface water anoxia when *Azolla* is cultivated for a longer time (Vroom et al. 2024) boosting soil-P release and subsequent P extraction. P extraction may further be enhanced through a combined cultivation with rooted macrophytes, such as *Typha* spp., that take up P directly from the soil. The presence of an emergent macrophyte could furthermore provide shade to *Azolla* and prevent growth limitation of *Azolla* by solar radiation (van Kempen et al. 2016).

As wet soil conditions can lead to increased emission of methane (CH_4_), assessing the effect of *Azolla* cultivation on GHG emissions is important. Raising the water table above the soil surface creates anoxic soil conditions, resulting in a transition from carbon dioxide (CO_2_) and nitrous oxide (N_2_O) dominated emissions (linked to respiration, CH_4_ oxidation, and, potentially, incomplete denitrification) to CH_4_ dominated emissions (methanogenesis) (Minke et al. 2016). Emissions of CH_4_, which has a 27 times higher global warming potential (GWP) than CO_2_ over a 100-year time horizon (Canadell et al. 2021), can be influenced by *Azolla* in various ways (Bodmer et al. 2024; Kosten et al. 2016; Vroom et al. 2022b). Briefly, an Azolla mat may reduce oxygen (O_2_) diffusion into the underlying water, but also CH_4_ diffusion from the water to the atmosphere. Furthermore, released organic matter (e.g. dead roots and root exudates) may provide a carbon source for methanogenesis. On the other hand, *Azolla* roots exhibit radial oxygen loss (ROL) and harbour CH_4_ oxidising bacteria, enhancing CH_4_ oxidation (Ávila et al. 2019). Lastly, the floating Azolla mat partly captures CH_4_ bubbles (ebullition), thereby increasing their residence time in the water and potential dissolution and/or oxidation. Besides CH_4_, nitrous oxide (N_2_O) is a GHG of concern, with a GWP of 273 on a 100-year time scale (IPCC 2019). N_2_O is a product of incomplete denitrification, and is formed in oxic conditions with high nitrate (NO_3_^−^) concentrations (Butterbach-Bahl et al. 2013). Although inundation is expected to negate N_2_O emissions by limiting soil O_2_ availability, the effect of N-fixing *Azolla* on N_2_O emissions remains unclear.

In this study, we assess the potential of *A. filiculoides* (hereafter: *Azolla*) cultivation for P extraction from inundated former agricultural soils. As *Azolla* can only take up P from surface water, we also assess the potential of a polyculture of *Azolla* and the rooted emergent macrophyte *Typha angustifolia* (hereafter: *Typha*). To this end, we performed an outdoor mesocosm experiment during two years using two different P-rich soils. We investigate 1) the effect of seasonality and other external factors on *Azolla* and *Typha* growth and P extraction, 2) how cultivation of these plants affects surface water and pore water composition, and 3) how *Azolla* and *Typha* cultivation affects emissions of the GHGs CH_4_ (diffusive and ebullitive) and N_2_O (diffusive). We hypothesise that *Azolla* growth rates and P sequestration are highest during the summer, as a result of high temperatures and sufficient sunlight. We expect that P sequestration rates will be higher in the *Azolla* + *Typha* polyculture due to additional porewater P uptake by *Typha* and enhanced *Azolla* growth due to protection from high solar radiation. We hypothesise that surface water nutrients will be lower in vegetated mesocosms than in unvegetated controls, and that surface water O_2_ concentrations will decrease after several months due to decaying organic matter input from vegetation. As a result of this input and reduced O_2_ concentrations, and due to the development of an anaerobic soil microbial community, we expect that CH_4_ emissions increase in time in vegetated mesocosms. In the polyculture, we expect low (surface water) diffusive and ebullitive CH_4_ emissions due to substantial plant CH_4_ transport by *Typha*. Lastly, we expect that N_2_O emissions in all treatments will only be high directly after inundation, because plant uptake, denitrification, and O_2_ limitation will quickly result in low surface water NO_3_^−^ concentrations.

## Materials and methods

### Soil collection

Soils were collected from two locations: ‘Allemanskamp’ (52°03′27"N 5°33′50"E) and ‘Leegveld’ (51°24′31"N 5°51′23"E). Both soils were classified as loamy fine sands. Hereafter, Allemanskamp soil is referred to as “ExP” (extreme P) and Leegveld soil as “HiP” (high P), based on their P availability (see Results Section “Soil characteristics”). At the time of sampling (18 June 2021), the ExP soil was still in agricultural use (corn production) whereas in Leegveld, agriculture had ceased recently (the last potato harvest took place less than a year before soil collection). Both soils had a high PO_4_^3−^ mobilization potential and were found suitable for *Azolla* cultivation based on a previous study (Vroom et al. 2024). After removing any present vegetation, the topsoil (upper 20 cm) was collected from both sites with an excavator, loaded onto a truck, and transported to the Radboud greenhouse facilities where it was stored at ambient temperature from 11 June until 21 June 2021.

### Experimental setup

**Fig. 1:**
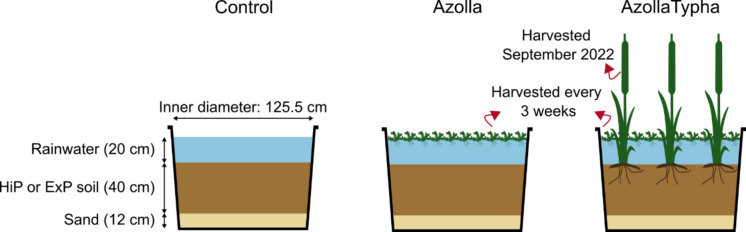
Overview of the experimental setup. For each soil (n = 2) and vegetation treatment (n = 3), four replicates were used (total n = 24 mesocosms)

The experiment consisted of 24 mesocosms installed outdoors at the Radboud University experimental garden. The facility is situated in an oceanic climate (*Cfb*) with a mean annual air temperature of 10.5 °C and annual precipitation of 851 mm (1991–2020, Koninklijk Nederlands Metereologisch Instituut). Each mesocosm consisted of a polyethylene tub with a volume of 750 L (121 cm inner diameter at the top, 82 cm height) which was embedded in the soil to limit temperature fluctuations. On 21 June 2021, mesocosms were filled with 12 cm of nutrient-poor drift sand (collected from 52°41′46.94"N, 4°38′11.22"E, chemical composition in table S1), followed by 40 cm of either ExP (n = 12) or HiP (n = 12) soil, to simulate a nutrient-rich top soil. From each mesocosm, a soil sample was collected into an air-free plastic bag and stored at 4 °C until further analyses. Then, soils were rewetted by gently adding rainwater up to 20 cm above the soil level. After inundation, mesocosms were loosely covered with opaque plastic covers to prevent algae growth but still allow gas exchange. After one month of stabilization, on 28 July 2021, three different vegetation treatments were applied (Fig. 1). In four mesocosms per soil (n = 8 mesocosms in total), three shoots of *Typha angustifolia* were planted. *T. angustifolia* shoots were collected from a nearby urban ditch (51° 52.06’ N, 5° 52.13’ E) and cut down to a standard length of 30 cm. Additionally, these mesocosms were inoculated with 900 g of fresh *Azolla filiculoides* (covering about 50% of the water surface), which was cultivated in the Radboud University greenhouse (“AzollaTypha” treatment). Four additional mesocosms per soil (8 mesocosms in total) were only inoculated with 900 g fresh *Azolla* (“Azolla” treatment). The eight remaining mesocosms were left unvegetated (“control” treatment). The experiment ran from June 2021 until November 2022.

Due to infestations with *Stenopelmus rufinasus* (Azolla weevil), all *Azolla* biomass was harvested in November 2021, re-introduced at 750 g fresh weight in April 2022, and then fully harvested again in September 2022. The water table was manually kept around 20 cm above the soil surface, which was marked at 10 cm below the mesocosm edge. Each week, either rainwater was added or excess water was gently scooped out with a bucket (taking care not to remove *Azolla* and preventing overflowing) when necessary. To prevent macro-ion limitation of *Azolla* due to the use of rainwater instead of surface water (which would most likely be used at a larger field scale), we added 500 µmol L^−1^ magnesium sulphate (MgSO_4_), 1000 µmol L^−1^ calcium chloride dihydrate (CaCl_2_·2H_2_O), and 500 µmol L^−1^ potassium chloride (KCl) on 28 July and 17 September 2021. Additionally, we supplied 500 µmol L^−1^ potassium sulphate (K_2_SO_4_) on 30 May, 29 June, and 19 August 2022. These moments were based on measured surface water K and S concentrations (see below).

### Soil analyses

To determine bio-available nutrient concentrations, extractions were carried out using 17.5 g of fresh soil, incubated with 50 mL of 0.2 M sodium chloride (NaCl, so-called ‘salt extractions’), after which pH of the supernatant was determined (for details, see Tomassen et al. 2004). Dry weight and bulk density were calculated after drying subsamples of a known volume of fresh soil at 70 °C for 48 h. Bio-available P (Olsen-P) was measured after incubating 3 g dried soil in 60 mL 0.5 M sodium carbonate (NaHCO_3_) for 30 min (Henriksen 1965). Amorphous aluminium (Al) and iron (Fe) and Fe/Al-bound P were determined after 2 h incubation with 50 mL of 0.11 M ammonium oxalate monohydrate and 0.9 M oxalic acid dehydrate, and fresh soil material equal to 5 g DW. All soil incubations were carried out on a shaker at 105 RPM, followed by fluid extraction using Rhizon samplers (Rhizosphere Research Products B.V.) under vacuum. Extracts were stored at −20°C until further analysis. The degree of soil phosphorus saturation (DPS; in %) was calculated according to Van der Zee et al. (1990):

$$DPS=\frac{{P}_{ox}}{0.5*({Al}_{ox}+{Fe}_{ox})}*100\%$$

where P_ox_, Al_ox_ and Fe_ox_ are the concentrations of amorphous P, Al, and Fe, respectively, obtained from oxalate extractions (mmol kg DW^−1^). PO_4_^3−^ mobilization rates were determined by calculating the slope of the linear increase in surface water PO_4_^3−^ for each mesocosm in the first month after inundation (prior to *Azolla* inoculation).

We calculated an indicative duration to reduce soil Olsen-P concentrations to 1000 µmol L^−1^ FW using *Azolla* cultivation (in optimal conditions) by dividing soil P surplus (kg ha^−1^) by *Azolla* P sequestration rate (kg P ha^−1^ yr^−1^) for each soil. Soil P surplus was calculated based on the ratio between soil total P and Olsen-P:

$${P}_{Surplus}=\left(TP- {P}_{Olsen, t1}* \frac{TP}{{P}_{Olsen, t0}}\right)*V$$

where TP is total soil P in kg L^−1^ FW, P_Olsen,t1_ is the target Olsen-P concentration in kg L^−1^ FW (equal to 1000 µmol L^−1^), P_Olsen,t0_ is the start Olsen-P concentration in kg L^−1^ FW, and V is the volume of topsoil (0–20 cm depth) in L ha^−1^.

### Water sampling & analyses

Surface water O_2_ concentrations and pH were determined in the upper 5 cm (*Azolla* root zone) using a Hach HQ40D portable multi-meter (Hach, Loveland, CO, USA) approximately every three weeks (every six weeks in winter). Surface water and pore water samples were taken in the same weeks. Surface water samples were taken from the upper 5 cm using a syringe. Pore water was extracted from the upper 10 cm of soil using ceramic cups attached to 60 mL syringes under vacuum. Directly after sampling, we measured green algae and cyanobacteria Chlorophyll (Chl) *a* in the surface water using a PHYTO-PAM phytoplankton analyser (Heinz Walz GmbH, Effeltrich, Germany). Water samples were split into two subsamples of which one was stored at 4 °C (after adding 0.1 mL of 65% HNO_3_ to a 10 mL sample) and the other at −20 °C until further analyses.

To determine dissolved CH_4_ concentrations, additional water samples were collected in gastight evacuated 12 mL glass exetainers (Labco, Lampeter, UK) containing 1 mL of 0.1 M hydrogen chloride (HCl) for sample preservation. Surface water was taken by puncturing a pre-evacuated exetainer with a needle that was submersed into the upper 5 cm surface water, while porewater samples were taken using the same ceramic cups as above. 5 to 10 mL water was collected, to leave sufficient headspace. After placing the exetainers horizontally on a shaker at 100 rpm for 60 min to allow headspace equilibration, dissolved CH_4_ was measured in the headspace of the exetainers using an HP 5890 gas chromatograph equipped with a Porapak Q column (80/100 mesh) and a flame ionization detector (GC-FID, Hewlett Packard, Palo Alto, CA, USA). Dissolved CH_4_ concentrations were then calculated using Henry's law (Sander 2015).

To gain insight in the maximum water demand of *Azolla* and *Azolla*/*Typha* cultivation, we temporarily stopped irrigation from 12 to 15 August 2022, when maximum day temperatures exceeded 30 °C. We calculated water loss due to evapotranspiration by measuring the distance from the edge of the mesocosm to the water surface using a ruler at the start and end of this period. Evapotranspiration in L m^−2^ d^−1^ was then calculated for each mesocosm using the water level difference.

### Methane flux measurements

In the same week as the water sampling, prior to *Azolla* harvesting, CH_4_ diffusion was measured using an opaque floating acrylic chamber (29.2 cm inner diameter) connected with gastight tubing (0.4 cm diameter) to an ultraportable greenhouse gas analyser (UGGA; ABB – Los Gatos Research, San Jose, CA, USA) in a closed, gastight circuit. Measurements were carried out during daytime and lasted 180s. The floating chamber was connected to two sticks that leaned on the edges of the mesocosms, to prevent disturbance due to movements, and to minimize the insertion depth in the water column/*Azolla* mat (< 2 cm insertion). If ebullition took place during the measurement (i.e. we observed a sudden steep increase in CH_4_ concentration), the measurement was repeated in a different place in the mesocosm. CH_4_ diffusion through *T. angustifolia* stems was measured separately. When stems were small, the abovementioned floating chamber was placed over a single stem, and the CH_4_ flux through the stem was calculated by subtracting the open water flux. When stems were larger (> 30 cm), an opaque PVC cylinder (60 or 130 cm), connected to the UGGA by tubing at the top, was placed over an individual stem. This cylinder was sealed from the water layer using a cap with a hole, which was plugged into a flexible sealing compound (Terostat IX; Teroson, Henkel, Germany) around the Typha stem. For 7 June 2022, an estimation of *T. angustifolia* contribution to total diffusive CH_4_ fluxes was made by multiplying the number of stems per m^2^ with the emission per stem.

During each CH_4_ measurement, air temperature in the chamber was logged every 30 s by a HOBO temperature logger (HOBO onset, Onset Computer Corporation, Bourne, MA, USA). CH_4_ fluxes were calculated according to Almeida et al. (2016) using the following equation:

$$F=\frac{V}{A}*\frac{dC}{dt}*\frac{P*M*F1}{R*T}$$

where F is the gas flux (mg m^−2^ d^−1^), V is chamber volume (m^3^), A is chamber surface area (m^2^), dC/dt is the change of the measured CH_4_ concentration over time (ppm s^−1^), P is the atmospheric pressure (atm), M is the molecular mass of CH_4_ (16.04 g mol^−1^), F1 is a conversion factor of seconds to days (86400), R is the gas constant (0.082057 L atm^−1^ K^−1^ mol^−1^), and T is atmospheric air temperature (K). Quality of the measured flux was assessed using R^2^ values of the linear regression (dC/dt). If R^2^ was below 0.9, fluxes were inspected visually. If the R^2^ was low because the flux was negligible (i.e. only noise), the flux was set to 0 mg m^−2^ d^−1^. If the flux was high, but R^2^ low due to excessive noise (e.g. due to ebullition), the measurement was excluded from the analyses.

To measure CH_4_ ebullition, we installed a bubble trap in each mesocosm consisting of a glass tube connected to an inverted plastic funnel (diameter 20 cm) which was attached with sticks to the mesocosm edges to ensure that the funnels were completely submerged and the traps were immobile (Fig. S1). A 1 cm thick butyl stopper was fitted on top of each glass tube to prevent gas loss while enabling gas extraction with a syringe. Gas volume was determined every 1.5–3 weeks by removing the accumulated gas with a syringe. Every 3–6 weeks, we flushed a 3 mL glass exetainer (Labco, Lampeter, UK) with the extracted gas. We then measured CH_4_ concentrations in a 30 µL subsample using an HP 5890 gas chromatograph as described above. Ebullitive CH_4_ fluxes were calculated by multiplying the volume with the CH_4_ concentrations. For dates on which only the gas volume was measured, CH_4_ concentration was calculated by linear interpolation. Bubble traps were removed during winter (December 2021 – March 2022), when ebullition was much decreased due to the low temperatures, to prevent frost damage to the glass bottles.

### Biomass sampling and analyses

*Azolla* was harvested manually every three weeks during the growing season. In 2021, all biomass was removed and placed in buckets with holes for 30 min to drain excess water. After determining fresh weight (FW), 750 g was separated and gently placed back into each mesocosm. In 2022, to further reduce handling stress, half of the *Azolla* surface was harvested and weighed, whereas the other half was evenly spread over the entire mesocosm surface. From each harvest, a subsample of 100 g fresh weight was dried at 70 °C for four days. *T. angustifolia* was harvested in 2022 only, to allow successful establishment. In September 2022, stems were cut just above the water table. Stems with and without inflorescences were separated and weighed. Inflorescences and subsamples of ten stems per mesocosm were dried separately. After drying, dry weight (DW) was determined of *Azolla* and *Typha* samples. *Typha* samples were first chopped to smaller pieces in a cutting mill (Retsch GmbH, Haan, Germany). Both *Azolla* and *Typha* samples were then ground using a ball mill (Retsch GmbH, Haan, Germany). Total phosphorus (TP) and total potassium (TK) contents were determined by digesting 200 mg dried plant material in 4 mL HNO_3_ (65%) and 1 mL H_2_O_2_ (35%) in Teflon vessels, heated in an Ethos D microwave (Milestone, Sorisole Lombardy, Italy). After digestion, samples were stored at 4 °C until chemical analysis. Total nitrogen (TN) and total carbon (TC) were measured by an elemental CNS analyser (NA 1500, Carlo Erba; Thermo Fisher Scientific, Franklin, USA) using 3 mg dried plant material.

### Chemical analyses

NH_4_^+^, NO_3_^−^, and PO_4_^3−^ concentrations were determined by colorimetric methods (Auto Analyser III, Bran and Luebbe GmbH, Norderstedt, Germany) in the water samples that were stored at −20 °C (Geurts et al. 2008). Inductively coupled plasma emission spectrometry (ICP-OES) was used to measure concentrations of Al, Fe, Mn, P, and Zn (IRIS Intrepid II, Thermo Electron corporation, Franklin, MA, USA) in the samples that were stored at 4 °C (water samples) and in the extraction and digestion samples.

### Statistical analyses

All statistical analyses and visualisations were done in R Studio (R version 4.3.2), using packages ggplot2 for data visualisation, nlme for linear mixed models, emmeans for post-hoc comparisons, and plyr for data exploration. Student’s t tests were used to determine differences in soil characteristics. Differences in variables measured over time (*Azolla* biomass production, surface water O_2_, green algae and cyanobacteria Chl* a*, nutrient concentrations in pore water and surface water, and CH_4_ concentrations and emissions) were assessed using linear (mixed) models, with treatment, soil, date and their two-way interactions as fixed factors. To account for repeated measurements, we added mesocosm as a random intercept and/or an auto-regressive moving average (ARMA) variance structure to correct for temporal auto-correlation, if this significantly lowered AIC values (tested with analysis of variance (ANOVA)). ANOVAs followed by Tukey post hoc tests were performed on the selected models to assess differences between individual treatments and dates. Model assumptions of t tests and linear (mixed) models were verified by visual examination of residual plots. Variables green algae Chl* a*, cyanobacteria Chl* a*, surface water NH_4_^+^, diffusive CH_4_ fluxes, and surface water and porewater dissolved CH_4_ showed a right-skewed residual distribution and were therefore log transformed to obtain normal distribution of residuals. A significance threshold of *p* < 0.05 was used in all cases. In-text values are reported as mean ± standard error.

## Results

### Soil characteristics

Both soils were very rich in P (P-Olsen > 2.5 mmol L^−1^), but ExP soil was more P-rich than HiP soil, illustrated by twice as high P concentrations in NaCl and oxalate extractions, a twice as high DPS, P mobilization rate, and P surplus, and almost five times higher porewater P concentrations (Table 1). Organic matter content was 1.5 times higher in HiP soil than ExP soil. Both soils had a similar pH (around 5.3) in NaCl extracts and the same low porewater Fe:P ratio (around 0.25).

**Table 1:** Characteristics of the soils used in the mesocosms (ExP = ‘Allemanskamp’, HiP = ‘Leegveld’)

Variable	Unit	ExP	HiP
OM	%	7.45 ± 0.15	11.15 ± 0.1***
Bulk density	kg L FW^−1^	0.86 ± 0.03	0.77 ± 0.02*
C	%	3.57 ± 0.13	5.51 ± 0.1***
N	%	0.34 ± 0.01	0.31 ± 0.01*
pH (NaCl extract)	-	5.32 ± 0.08	5.29 ± 0.05
NaCl-extractable N	µmol kg^−1^ DW	2021 ± 741	1373 ± 380*
NaCl-extractable P	µmol kg^−1^ DW	354.7 ± 11.8	180 ± 5.6*
NaCl-extractable Fe	µmol kg^−1^ DW	6.08 ± 0.28	6.6 ± 0.24
P-Olsen	mmol L^−1^ FW	3.69 ± 0.12	2.71 ± 0.06***
Oxalate-extractable Al	mmol kg^−1^ DW	21.5 ± 0.31	34.96 ± 0.39***
Oxalate-extractable Fe	mmol kg^−1^ DW	31.84 ± 1.09	16.05 ± 0.23***
Oxalate-extractable P	mmol kg^−1^ DW	32.71 ± 1.08	15.27 ± 0.14***
Porewater P	µmol L^−1^	1440 ± 147	301 ± 11***
Porewater Fe	µmol L^−1^	354.1 ± 37.5	74.3 ± 2.8***
Porewater Fe:P	-	0.246 ± 0.01	0.247 ± 0.004
DPS	%	122.5 ± 1.2	59.9 ± 0.4***
TP	mmol kg^−1^ DW	40.9 ± 0.9	19.2 ± 0.4***
PO_4_^3−^ mobilisation rate	µmol m^−2^ d^−1^	924 ± 81	414 ± 11***
P surplus	kg ha^−1^	1706 ± 129	616 ± 40***

Values shown are average ± SE (n = 12 soil samples per soil). Porewater values were measured 28 days after inundation. Asterisks indicate significant differences between soils (* *p* < 0.05, ** *p* < 0.01, *** *p* < 0.001)

### Biomass production and P extraction

**Fig. 2:**
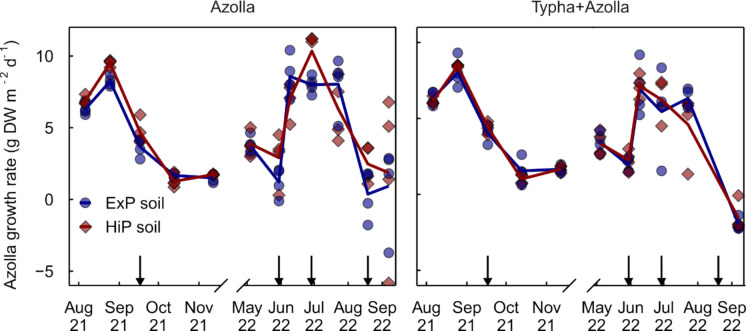
Absolute growth rates of *Azolla* in absence and presence of *Typha* throughout the two growth periods (Jul-Nov 2021 and Apr-Sep 2022). *Azolla* was harvested every three weeks from both treatments. Black arrows represent addition of macro-ions (Mg, SO_4_, and K in 2021, SO_4_ and K in 2022, see Section 2.2). Lines represent average trends, symbols represent individual mesocosms

*Azolla* growth rates peaked at the end of August 2021 (8.4 ± 0.3 g DW m^−2^ d^−1^ on ExP soil and 9.3 ± 0.1 g DW m^−2^ d^−1^ on HiP soil in both Azolla and AzollaTypha treatments) and decreased gradually in the following months until biomass was harvested due to *S. rufinasus* invasion in November (Fig. 2, Table S2). After re-introduction on 12 April 2022, growth rates were low until additional K_2_SO_4_ was added (mid-June). Growth rates then became high (> 5 g DW m^−2^ d^−1^) in most mesocosms, until the end of August, when *S. rufinasus* invaded anew and addition of water was stopped during a drought period, causing the water table in several AzollaTypha mesocosms to drop below soil surface. Both factors led to a decrease in *Azolla* growth rates, most prominently so in the AzollaTypha treatments. In 2021, biomass production was slightly higher on HiP soil than ExP soil in the Azolla treatment only (p = 0.002) (Table 2). This pattern was similar in 2022, with about 16% higher biomass production on HiP soil than ExP soil in the Azolla treatment only (*p* = 0.006). Biomass production was about 60% higher in Azolla treatments than in AzollaTypha treatments (*p* < 0.001). *Azolla* P content was almost twice as high on ExP soil (0.69 ± 0.02%) as on HiP soil (0.36 ± 0.02%) (*p* < 0.001). Only on ExP soil, P content differed between vegetation treatments, with higher P content in Azolla (0.72 ± 0.02%) than in AzollaTypha treatments (0.65 ± 0.02%) (*p* = 0.005). Total P sequestration was over 20% higher in 2022 than in 2021 (*p* = 0.002). There were no differences in P sequestration between vegetation treatments (*p* = 0.85) in 2021, but in 2022, P sequestration by Azolla was 1.4 times higher in Azolla than in AzollaTypha treatments (*p* < 0.001).

**Table 2:** Plant yield, P removal, and extrapolated P removal for both plant treatments and both soils during two years

			Azolla				Typha + Azolla			
			ExP soil		HiP soil		ExP soil		HiP soil	
	Variable	Unit	2021	2022	2021	2022	2021	2022	2021	2022
*Azolla*	Total yield	g DW m^−2^	386 ± 13	580 ± 41	435 ± 19	691 ± 40	421 ± 22	393 ± 32	424 ± 13	395 ± 8
	Growth rate	g DW m^−1^ d^−1^	3.61 ± 0.12	3.92 ± 0.28	4.07 ± 0.18	4.67 ± 0.27	3.94 ± 0.21	2.65 ± 0.21	3.96 ± 0.12	2.67 ± 0.05
	P content	%	0.68 ± 0.03	0.76 ± 0.01	0.39 ± 0.04	0.3 ± 0.01	0.61 ± 0.02	0.69 ± 0.02	0.41 ± 0.02	0.34 ± 0.01
	N content	%	7.39 ± 0.14	4.66 ± 0.63	10.42 ± 0.38	4.61 ± 0.55	7.83 ± 0.22	4.5 ± 0.51	9.81 ± 0.07	4.48 ± 0.52
	N:P quotient	-	10.9 ± 0.43	6.14 ± 0.83	27.44 ± 3.36	15.13 ± 1.46	12.95 ± 0.47	6.54 ± 0.81	23.88 ± 1.35	13.53 ± 1.87
	Net P removal	g m^−2^	2.6 ± 0.06	3.83 ± 0.4	1.57 ± 0.09	1.96 ± 0.1	2.51 ± 0.13	3.06 ± 0.25	1.59 ± 0.05	1.18 ± 0.07
	Net P removal	kg ha^−1^	26.01 ± 0.63	38.28 ± 3.99	15.68 ± 0.95	19.6 ± 1.04	25.11 ± 1.29	30.58 ± 2.54	15.88 ± 0.55	11.76 ± 0.69
*Typha*	Total yield	g DW m^−2^						1187 ± 43		1151 ± 27
	Growth rate	g DW m^−1^ d^−1^						2.9 ± 0.1		2.8 ± 0.1
	P content	%						0.31 ± 0.02		0.27 ± 0.01
	N content	%						1.72 ± 0.08		1.24 ± 0.07
	N:P quotient	-						5.59 ± 0.16		4.65 ± 0.12
	Net P removal	g m^−2^						3.67 ± 0.33		3.06 ± 0.06
	Net N removal	g m^−2^						20.44 ± 1.58		14.26 ± 0.57
	Extrapolated P removal	kg ha^−1^						36.68 ± 3.29		30.64 ± 0.65
	Extrapolated N removal	kg ha^−1^						204.4 ± 15.8		142.6 ± 5.7
Total	Extrapolated P removal	kg ha^−1^ yr^−1^	26 ± 0.6	38.3 ± 4	15.7 ± 0.9	19.6 ± 1	25.1 ± 1.3	67.3 ± 5.1	15.9 ± 0.5	42.4 ± 1
	Estimated P extraction duration	yr	63.3 ± 5.4	45 ± 7.1	39.9 ± 4.6	31.9 ± 3.7	76.1 ± 14	27.9 ± 4.2	33.1 ± 1.5	12.4 ± 0.7

Values are averages ± standard error and are based on all sampling moments (*T. angustifolia* was only harvested in 2022). Extrapolated P removal is based on a growing season of 3.5 months (107 days) in 2021 and 4 months (126 days) in 2022. Estimated P extraction duration is based on a target concentration of 1000 µmol L^−1^ Olsen-P

*T. angustifolia* expanded rapidly in 2022, with similar biomass production (1.17 ± 0.02 kg DW m^−2^ after two growing seasons; *p* = 0.50) and P content (0.09 ± 0.01%; *p* = 0.07) on both soils. P removal by *Typha* after two growing seasons was therefore also similar on both soils, at 3.37 ± 0.19 g m^−2^ (33.7 ± 1.9 kg ha^−1^; *p* = 0.12).

In controls, we observed algae growth in four mesocosms (two for each soil type) (see Chl *a* measurements below). The four remaining control mesocosms experienced spontaneous colonisation by macrophytes, predominantly by *Typha latifolia*.

### Water chemistry

**Fig. 3:**
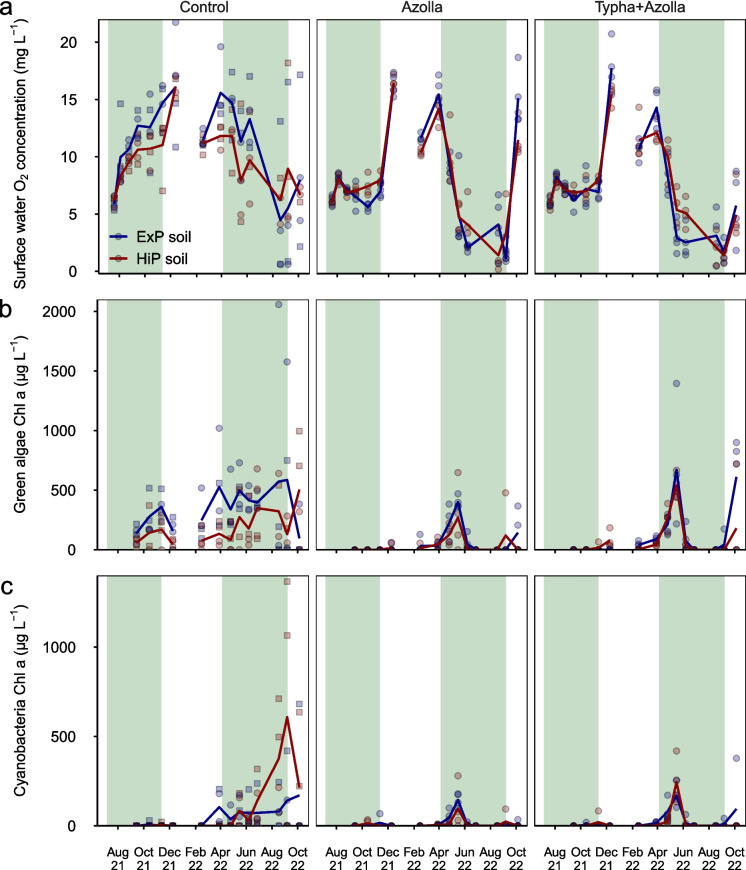
Surface water O_2_ **a** and phytoplankton Chl* a* **b**, **c** concentrations throughout the experiment. Lines represent average trends, symbols represent individual mesocosms. In controls, square symbols represent algae-dominated mesocosms while circles represent macrophyte-dominated mesocosms. Green shaded areas are periods in which *Azolla* was present

In 2021, all controls were supersaturated with O_2_ during the day (11.1 ± 0.5 mg L^−1^), with higher concentrations than both vegetated treatments (*p* < 0.001), where the surface water remained oxic (8.4 ± 0.5 mg L^−1^) (Fig. 3a). In December, after *Azolla* removal, vegetated treatments attained similar O_2_ concentrations as the controls. In 2022, O_2_ concentrations were similar in all treatments until *Azolla* was re-introduced in April. After re-introduction, O_2_ concentrations in vegetated treatments became lower than controls (3.1 ± 0.4 mg L^−1^ in May through September, *p* < 0.04). In some mesocosms, hypoxia was observed (< 2 mg O_2_ L^−1^) during the summer months. Finally, after *Azolla* removal, O_2_ concentrations in October increased again in both vegetated treatments, reaching higher concentrations in Azolla than in AzollaTypha (*p* < 0.001) and control (*p* = 0.004) treatments.

In 2021, green algae Chl* a* concentrations were highest in the controls (*p* < 0.001), with twice higher concentrations on ExP soils (234 ± 36 µg L^−1^) than HiP soils (104 ± 28 µg L^−1^) (*p* = 0.002) (Fig. 3b). No green algae Chl *a* was observed in vegetated treatments in September – November, with the exception of low concentrations (< 20 µg L^−1^) in AzollaTypha treatments on HiP soil in November. After *Azolla* removal in November 2021, green algae Chl* a* concentrations rose gradually in vegetated treatments until reaching similar concentrations to controls in March 2022. In June, when *Azolla* cover was re-established, concentrations in vegetated treatments became negligible again until *Azolla* was removed, with no differences between treatments in October. In 2022, overall green algae Chl* a* concentrations were approximately twice as high on ExP soils than on HiP soils (*p* = 0.04).

Cyanobacteria concentrations were negligible (< 20 µg L^−1^) in 2021 (Fig. 3c). Concentrations gradually rose in the beginning of 2022. In June, concentrations became significantly lower in vegetated treatments than in controls (*p* < 0.03) until the end of the experiment. At the end of June and in August, cyanobacteria Chl *a* was zero in all vegetated treatments. There were no differences between soils (*p* = 0.21).

**Fig. 4:**
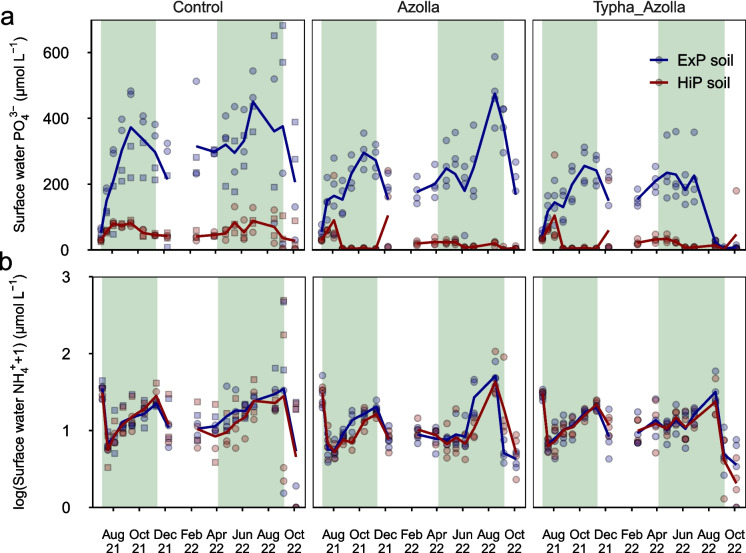
Surface water PO_4_^3−^ **a** and NH_4_^+^ **b** concentrations throughout the experiment. Lines represent average trends, symbols represent individual mesocosms. In controls, square symbols represent algae-dominated mesocosms while circles represent macrophyte-dominated mesocosms. Green shaded areas are periods in which *Azolla* was present. NB: NH_4_^+^ concentrations are on a logarithmic scale

Surface water PO_4_^3−^ concentrations were about six times higher on ExP soils (226 ± 10 µmol L^−1^) than HiP soils (37 ± 3 µmol L^−1^) (*p* < 0.001) (Fig. 4a). Concentrations were lower in vegetated treatments than in controls (*p* < 0.001), with no differences between Azolla and AzollaTypha treatments (*p* = 0.11). In August 2022, the heatwave-induced water table drop caused a steep decrease in PO_4_^3−^ concentrations. Surface water NO_3_^−^ concentrations were substantial (~ 100 µmol L^−1^) in the beginning of the experiment, and significantly higher on ExP than HiP soils (*p* = 0.004) (Fig. S2). Concentrations dropped steeply within two months after inundation, becoming negligible (< 10 µmol L^−1^) in all treatments until the end of the experiment. Similar to NO_3_^−^, surface water NH_4_^+^ concentrations showed an initial peak, then dropped to low (< 10 µmol L^−1^) concentrations three weeks after inundation (Fig. 4b). Surface water NH_4_^+^ concentrations showed some seasonal variation, but were not influenced by soil type (*p* = 0.24) or plant treatment. In September 2022, after the drought event, concentrations peaked briefly, especially in algae-dominated controls.

**Fig. 5:**
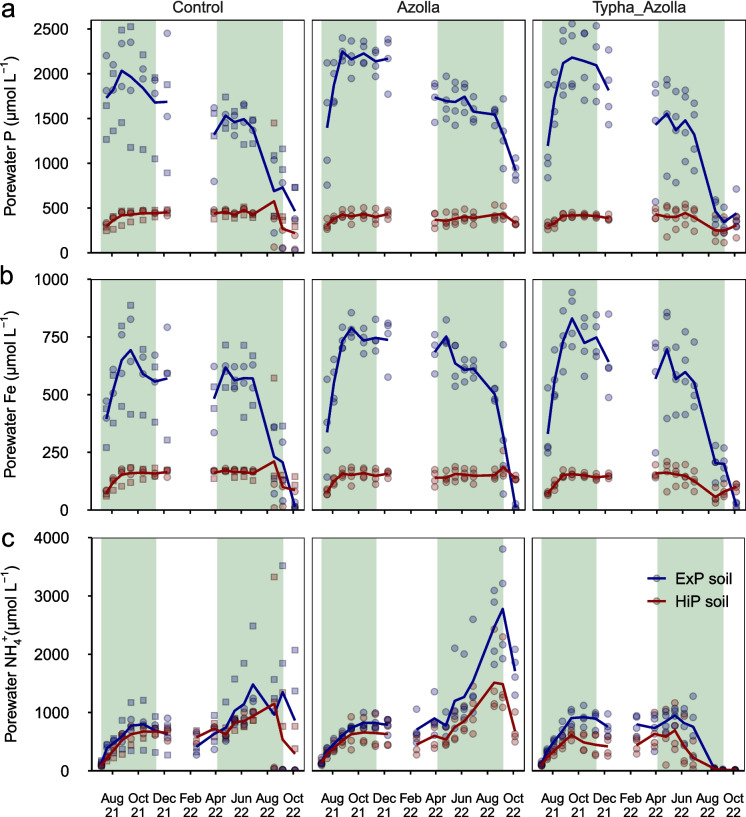
Pore water P **a**, Fe **b** and NH_4_^+^ **c** concentrations throughout the experiment. Lines represent average trends, symbols represent individual mesocosms. In controls, square symbols represent algae-dominated mesocosms while circles represent macrophyte-dominated mesocosms. Green shaded areas are periods in which *Azolla* was present

Overall, porewater P concentrations were about four times higher in ExP soils (1553 ± 44 µmol L^−1^) than in HiP soils (389 ± 9 µmol L^−1^) (*p* < 0.001), with no significant differences between treatments (*p* = 0.09) (Fig. 5a). After the drought event in August, P concentrations dropped steeply in all treatments. Porewater Fe concentrations showed a similar pattern to porewater P, with nearly four times higher concentrations in ExP soils (532 ± 18 µmol L^−1^) than in HiP soils (140 ± 4 µmol L^−1^) (*p* < 0.001) and no differences between treatments (*p* = 0.18) (Fig. 5b). A steep drop in Fe concentrations was also observed in August 2022. Porewater NH_4_^+^ concentrations were slightly higher in ExP soils (806 ± 45 µmol L^−1^) than HiP soils (570 ± 30 µmol L^−1^) (Fig. 5C). In August and September, concentrations were highest in Azolla treatments, followed by controls, and Typha treatments, respectively (*p* < 0.002).

Water loss due to evapotranspiration was highest in AzollaTypha mesocosms (29.9 ± 0.7 L m^−2^ d^−1^), followed by the controls dominated by *T. latifolia* (20.5 ± 6.2 L m^−2^ d^−1^), which had less dense stands. Azolla treatments (5.4 ± 0.7 L m^−2^ d^−1^) and algae-dominated controls (5.3 ± 0.9 L m^−2^ d^−1^) had similar and much lower evapotranspiration rates (Fig. S3).

### GHG emissions

**Fig. 6:**
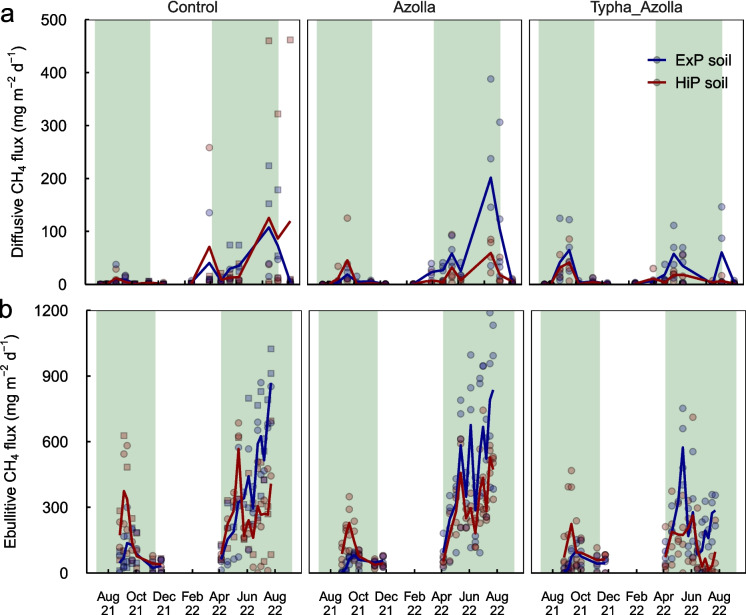
Diffusive **a** and ebullitive **b** CH_4_ emissions throughout the experiment (NB: these values do not include CH_4_ transport through Typha plants and bubble traps were placed below the *Azolla* mat). Lines represent average trends, symbols represent individual mesocosms. In controls, square symbols represent algae-dominated mesocosms while circles represent macrophyte-dominated mesocosms. Green shaded areas are periods in which *Azolla* was present

Diffusive CH_4_ fluxes (excluding fluxes through *T. angustifolia*) were negligible (0.4 ± 0.1 mg m^−2^ d^−1^) in the first two months after rewetting, after which emissions increased, then became negligible again during the winter (Fig. 6a). From March on, emissions rose again and generally peaked during August 2022 (mostly staying below 100 mg m^−2^ d^−1^). Overall, emissions were slightly higher on ExP soil (27.2 ± 4.0 mg m^−2^ d^−1^) than HiP soil (20.1 ± 5.2 mg m^−2^ d^−1^) (*p* < 0.001). There were no significant differences between vegetation treatments (*p* = 0.15).

CH_4_ ebullition was substantial, with fluxes up to 1189 mg m^−2^ d^−1^, and varied widely within treatments and between dates (Fig. 6b). CH_4_ concentrations in the sampled gas varied between 0 and 79.6%, with an average of 45.5% (standard deviation 23.5%). CH_4_ ebullition was higher in 2022 than in 2021 and peaked during the summer months. Ebullitive CH_4_ emissions were higher on HiP soil than ExP soil in September 2021 (*p* < 0.008), but higher on ExP soil than HiP soil in June and July 2022 (*p* < 0.031). Treatment differences showed lower CH_4_ ebullitive flux in AzollaTypha treatments than control and Azolla treatments in June and July 2022 (*p* < 0.005).

Surface water CH_4_ concentrations were low (< 5 µmol L^−1^) in 2021, and increased in 2022, staying mostly below 40 µmol L^−1^ (Fig. S4a). In June 2022, concentrations were higher in controls than in Azolla and AzollaTypha treatments (*p* < 0.001). In August 2022, concentrations were lower in the AzollaTypha treatment than in the controls (*p* < 0.001) with no further differences between treatments. There were no differences between soils (*p* = 0.06). Porewater dissolved CH_4_ showed a gradual increase in 2021, and fluctuated around 800 µmol L^−1^ in 2022 (Fig. S4b). There were no differences between treatments until after the drought event, when concentrations in AzollaTypha treatments and macrophyte-dominated controls dropped. There were no significant differences between soils (*p* = 0.66).

**Fig. 7:**
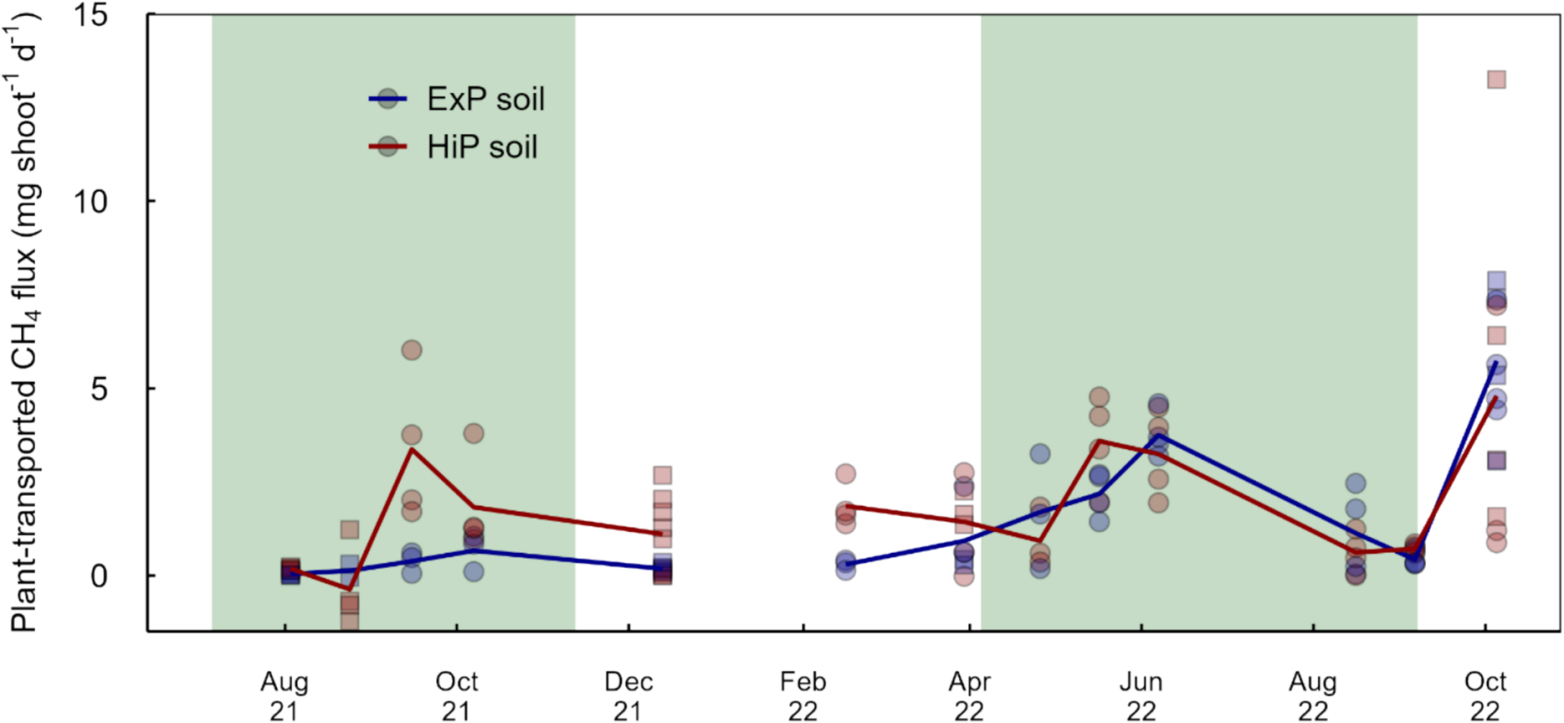
CH_4_ emitted through Typha shoots. Circles represent fluxes measured through living shoots, squares represent fluxes through dead shoots or stumps. Lines represent average trends, symbols represent individual mesocosms. Green shaded areas are periods in which *Azolla* was present. Fluxes from October 2022 were measured after Typha was harvested in September

CH_4_ fluxes through *T. angustifolia* averaged 1.7 ± 0.2 mg stem^−1^ d^−1^ and peaked during the summer seasons and in October 2022, after stems were harvested (Fig. 7). In June 2022, plant-transported CH_4_ emissions averaged 21.2 ± 3.55 mg m^−2^ d^−1^, contributing 20 to 96% (average 55 ± 12%) to total diffusive emissions.

N_2_O fluxes peaked in the first month after rewetting (Fig. S5), then remained below detection limits during the remainder of the experiment.

## Discussion

*Azolla* cultivation could be an effective means to extract P from former agricultural soils after inundation or rewetting, and could thereby aid in successful wetland rehabilitation or creation by preventing eutrophication. In this study, we evaluated this concept using 24 outdoor mesocosms with two P-rich former agricultural soils. We found that *Azolla* growth and P extraction rates were high during summer months, but growing season length was constrained by pest invasion, specifically of the weevil *S. rufinasus*. This constraint led to only moderate yearly P extraction rates. Polyculture with *Azolla* and *T. angustifolia* yielded similar amounts of *Azolla* biomass as the *Azolla* monoculture and led to higher yearly P extraction rates. Furthermore, we found that *Azolla* cover prevented microalgae and spontaneous emergent macrophyte growth. *Azolla* cover led to surface water anoxia in the 2nd year after inundation, potentially accelerating sediment P mobilization. *Azolla* cultivation reduced surface water PO_4_^3−^ concentrations, but this decrease was limited when surface water PO_4_^3−^ concentrations were extremely high (ExP soil). Ebullitive CH_4_ emissions were substantial and exceeded diffusive CH_4_ emissions, but emissions were not affected by *Azolla* cultivation. *T. angustifolia* cultivation resulted in lower peak ebullitive emissions, but CH_4_ emissions through plant shoots were substantial. N_2_O emissions were negligible, only occurring directly after soil inundation.

### P extraction by Azolla and Azolla-Typha cultivation

Maximum *Azolla* growth rates of around 8 g DW m^−2^ d^−1^ during the summer months were about twice as high as those previously recorded in a greenhouse experiment using the same soils (Vroom et al. 2024). Similar growth rates on both soils, but a much higher *Azolla* P content on ExP soil, indicates luxury consumption of P, which is typical for *Azolla* (Peeters et al. 2016; Temmink et al. 2018). If the *Azolla* growing season is, as in our case, limited by *S. rufinasus* invasion, P extraction of a hypereutrophic former agricultural soil down to 1000 µmol Olsen-P would take multiple decades. In absence of *S. rufinasus*, *Azolla* could survive year-round in a temperate climate, as it can withstand temperatures below 0 ⁰C and recover after being covered in ice for a week, and even retain positive growth rates during winter (Janes 1998). Successful (bio-)control of *S. rufinasus* could thus result in substantially higher P extraction rates.

### High P extraction rates, but methodological challenges, in Azolla-Typha polyculture

*T. angustifolia* yield and P removal were comparable to *T. latifolia* stands on rewetted peat and mineral soils, while P content was at the high end of the range (Geurts et al. 2020). N removal by *Typha* was moderate compared to these stands. The high *Typha* P content can be explained by the very high soil P content and optimal harvest timing in our experiment (Pijlman et al. 2019). High P extraction rates by *Azolla*-*Typha* polyculture (up to 67 kg ha^−1^ yr^−1^) indicate that it is an effective method for rapid P removal. Removal rates exceeded those of previously tested methods on similar soils, such as potassium-fertilized grass-clover swards that are regularly mowed (34 kg P ha^−1^ yr^−1^; Timmermans and van Eekeren 2016) or agricultural cultivation and mowing of N-fertilized grass sward (28–50 kg ha^−1^ yr^−1^; van der Salm et al. 2009). However, upscaling of this polyculture may prove challenging, firstly, because harvesting *Azolla* in-between *Typha* plants is challenging on a large scale. Secondly, high evaporation rates by *T. angustifolia* in summer require the supply of large amounts of water, especially since *Azolla* requires a water table above the soil surface.

As *Azolla* biomass yield in the polyculture was lower than in the monoculture in 2022, *Typha* may have competed with *Azolla* for light and/or space. Competition for nutrients was less likely, as nutrient concentrations were very high both in the porewater and surface water. As we found no negative effect (i.e. red coloration; Lumpkin and Plucknett 1980) of solar radiation on *Azolla* grown on its own, there was also no indication of *Typha* shading positively affecting *Azolla* growth. This lack of effect can be explained by the high nutrient availability in the water (Sadeghi et al. 2013). We found no evidence that *Azolla* supplied additional N to *Typha*, a concept used in rice cultivation (Xu et al. 2017), but any supply may have been obscured by the already high soil N mobilization, preventing N limitation in *Typha*. In the long term, as nutrient concentrations decrease due to harvesting, competition between the two species may occur (Vroom et al. 2018). Furthermore, a denser *Typha* stand might constrain *Azolla* growth by limiting light and space availability.

### Impact of Azolla cultivation on surface water quality

Low surface water O_2_ concentrations in Azolla treatments only in the second year of *Azolla* cultivation indicate that the combination of an *Azolla* mat and sufficient decaying organic matter led to anoxia, by limiting atmospheric O_2_ diffusion and by consuming O_2_, respectively. This mechanism is further supported by a steep increase in surface water O_2_ concentrations after *Azolla* removal, and corroborates earlier findings (Vroom et al. 2024). However, O_2_ measurements were only taken every three weeks and pertain to daytime O_2_ concentrations only, whereas nighttime concentrations may be substantially lower due to absence of photosynthesis.

When soils were not vegetated after inundation, they developed in two distinct ways: domination by green algae and cyanobacteria, preventing light intrusion and plant growth, or colonization by emergent macrophytes (in this case mostly *T. latifolia*). This finding shows that, when no action is taken after inundation of P-rich agricultural land, a system with low plant biodiversity will develop (Geurts et al. 2008).

As expected, *Azolla* cultivation lowered surface water P concentrations. However, there was a limit to this uptake, as concentrations on the ExP soil, with extremely high P content, remained high, even in mesocosms with *Typha*, presumably due to very high P mobilization rates. This mechanism is further supported by stable porewater N and P concentrations in the presence of *Typha*, despite uptake by the rooted vegetation (Vroom et al. 2022a). Limits to P uptake should be taken into account when upscaling to prevent losses of P downstream of the inundated site (Surridge et al. 2012).

### Greenhouse gas emissions of Azolla cultivation

Diffusive CH_4_ emissions were moderate, and comparable to those found in a nearby urban pond (27 mg m^−2^ d^−1^; van Bergen et al. 2019). Total CH_4_ emissions from all treatments were substantial, and dominated by ebullition. Ebullition-dominated CH_4_ emissions are characteristic for highly productive water bodies, where rates of methanogenesis are high (Flickinger et al. 2020; van Bergen et al. 2019). In shallow water bodies, bubbles are easily released because of low hydrostatic pressure and a short residence time in the surface water, limiting potential CH_4_ dissolution and oxidation (Bastviken 2009).

*Azolla* cover did not have a clear effect on diffusive or ebullitive CH_4_ emissions, despite causing surface water anoxia. However, as our bubble trap funnels were placed below the *Azolla* mat, physical retention of bubbles by the plants was not taken into account. In a previous study, it was found that *Azolla* can retain 10% and 43% of bubbles at 100 and 300% plant coverage, respectively (Kosten et al. 2016). Retained bubbles may dissolve or be oxidized in the plant root zone. Depending on the thickness of the mat (and thus the time after harvesting), root zone oxidation could substantially reduce ebullitive CH_4_ emissions. On the other hand, regular harvesting of the *Azolla* biomass likely results in the instant release of these retained bubbles.

Lower ebullitive CH_4_ emissions from the *Azolla*-*Typha* polyculture during the summer of 2022 can be explained by plant transport, decreasing the soil CH_4_ pool (van den Berg et al. 2020; Van Der Nat et al. 1998), and radial oxygen losses (ROL) to the soil by the *Typha* roots, reducing methanogenesis and enhancing CH_4_ oxidation (Aben et al. 2022). This explanation is supported by substantial CH_4_ emissions measured through individual *Typha* stems. CH_4_ transport may vary for each individual stem within a stand, for instance depending on stem age, belowground connectivity and root morphology (Vroom et al. 2022b). Furthermore, plant transport processes can be affected by daylight and temperature. These factors should be taken into account to capture the full effect of *Typha* transport on CH_4_ emissions.

N_2_O emissions observed in the first month after rewetting were probably caused by the initially high surface water (and soil) NO_3_^−^ concentrations and soil water saturation, leading to incomplete denitrification (Maltais-Landry et al. 2009). As expected, N_2_O emissions then dwindled as NO_3_^−^ was depleted. We can therefore conclude that N_2_O emissions are negligible soon after inundation of former agricultural soils (if no additional NO_3_^−^ loading takes place), also in the presence of *Azolla*.

### Implications and recommendations for future research

By *S. rufinasus* was the main constraint on *Azolla* growth in our experiment, posing a major challenge to applying this method on a larger scale. *S. rufinasus* has been recorded in North America, Europe, and South Africa. Prior to upscaling in these locations, future research should delve into potential biological eradication methods. For instance, we found in a pilot experiment that the natural insecticide pyrethrum (Raptol®), extracted from dried flowers of *Chrysanthemum cinerariifolium* and *Chrysanthemum coccineum*, successfully eradicated *S. rufinasus* and led to full recovery of the *Azolla* mat (Van de Riet et al., unpublished results). However, as pyrethrum can be toxic to macrofauna and fish (Mauck et al. 1976), it is essential to assess its release to the surface water after application. Additionally, it could be valuable to test biological control agents (e.g. parasitoid wasps, fungi, fish). In absence of *S. rufinasus*, *Azolla* could survive year-round in a temperate climate (Janes 1998). Year-round cultivation would result in much higher P extraction rates and thus a much shorter extraction period needed to reach P limited conditions. For instance, if *Azolla* grown on ExP soil would attain high growth rates and P content as seen in summer (8 mg DW m^−2^ d^−1^, 0.6% P) during six months a year, P removal would be 88 kg ha^−1^ yr^−1^, and extraction duration would decrease from 63 to 19 years. In conclusion, *Azolla* cultivation could be a viable and relatively fast means of P extraction when sufficient water is available, and with successful biological control of *S. rufinasus*. *Azolla* cultivation did not lead to elevated CH_4_ and N_2_O emissions compared to open water or emergent macrophytes. Extracted P could be recycled when using *Azolla* as a biofertilizer or *T. angustifolia* as an animal feed, reducing the need for mineral P mining. Future research should therefore investigate *Azolla* growth and P extraction potential year-round with the use of sustainable pest control measures.

## Supplementary Information

Below is the link to the electronic supplementary material.

Supplementary file1 (DOCX 1.18 KB)

## Data Availability

The data that support the findings of this study are openly available in Zenodo at 10.5281/zenodo.10634272.
