# Supplementary material for: Nutrient dynamics and GHG emissions in Azolla and Typha based cultivation on inundated former agricultural soils
Source: Plant Soil. 2025 Nov 17;518(1):819–38. doi: 10.1007/s11104-025-08032-y (PMC12830449; doi:10.1007/s11104-025-08032-y)
Supplement: Supplementary file 1 — Supplementary file1 (DOCX 1.18 KB) [file 11104_2025_8032_MOESM1_ESM.docx]

**Supplementary materials**

**Table S1** Chemical composition of the drift sand used as a 12 cm layer underneath the agricultural soils. Values are presented as average ± standard error (n=3 soil samples).

|  |  |  |
| --- | --- | --- |
| Variable | Unit | Sand layer |
| OM | % DW | 0.19 ± 0.03 |
| C | % | 0.26 ± 0.02 |
| N | % | 0 ± 0 |
| pH (NaCl extract) | - | 7.82 ± 0.2 |
| NaCl-extractable N | µmol kg DW^-1^ | 33.54 ± 6 |
| NaCl-extractable P | µmol kg DW^-1^ | 13.8 ± 1.3 |
| NaCl-extractable Fe | µmol kg DW^-1^ | 0.92 ± 0.04 |
| P-Olsen | mmol L FW^-1^ | 0.07 ± 0.01 |
| Oxalate-extractable Al | mmol kg DW^-1^ | 0.6 ± 0.01 |
| Oxalate-extractable Fe | mmol kg DW^-1^ | 2.17 ± 0.03 |
| Oxalate-extractable P | mmol kg DW^-1^ | 0.27 ± 0.01 |
| DPS | % | 19.38 ± 0.9 |
| TP | mmol kg DW-1 | 1.6 ± 0.23 |
|  |  |  |

**Table S2** *Azolla* growth rate and nutrient content throughout the two growing periods. Values are presented as average ± standard error (n=8 biomass samples per soil; n=5 biomass samples per soil for 19-8-2022).

|  |  |  |  |  |  |  |  |  |  |  |
| --- | --- | --- | --- | --- | --- | --- | --- | --- | --- | --- |
|  | ExP soil |  |  |  |  | HiP soil |  |  |  |  |
|  | Growth rate | P content | N content | Fe content | K content | Growth rate | P content | N content | Fe content | K content |
| Date | (mg m^-2^ d^-1^) | (%) | (%) | (%) | (%) | (mg m^-2^ d^-1^) | (%) | (%) | (%) | (%) |
| 06-08-2021 | 6.56 ± 0.18 | 0.6 ± 0.01 | 12.11 ± 0.16 | 0.047 ± 0.006 | 2.63 ± 0.06 | 6.74 ± 0.1 | 0.59 ± 0.11 | 12.54 ± 0.12 | 0.04 ± 0.002 | 3.49 ± 0.07 |
| 25-08-2021 | 8.43 ± 0.26 | 0.65 ± 0.03 | 11.21 ± 0.52 | 0.044 ± 0.004 | 1.16 ± 0.06 | 9.31 ± 0.09 | 0.3 ± 0.09 | 12.94 ± 0.26 | 0.04 ± 0.002 | 1.65 ± 0.07 |
| 17-09-2021 | 3.99 ± 0.24 | 0.62 ± 0.05 | 13.21 ± 0.48 | 0.112 ± 0.009 | 0.82 ± 0.03 | 4.71 ± 0.21 | 0.31 ± 0.22 | 17.03 ± 0.28 | 0.08 ± 0.005 | 1.17 ± 0.07 |
| 13-10-2021 | 1.67 ± 0.21 | 0.6 ± 0.02 | 2.76 ± 0.21 | 0.177 ± 0.029 | 0.91 ± 0.04 | 1.18 ± 0.13 | 0.46 ± 0.13 | 2.43 ± 0.05 | 0.09 ± 0.004 | 1.5 ± 0.07 |
| 12-11-2021 | 1.64 ± 0.11 | 0.71 ± 0.03 | 3.38 ± 0.08 | 0.212 ± 0.039 | 1.3 ± 0.04 | 1.77 ± 0.05 | 0.44 ± 0.05 | 3.41 ± 0.03 | 0.07 ± 0.004 | 1.7 ± 0.04 |
|  |  |  |  |  |  |  |  |  |  |  |
| 04-05-2022 | 3.72 ± 0.22 | 0.69 ± 0.04 | 12.92 ± 0.31 | 0.22 ± 0.035 | 1.34 ± 0.08 | 3.74 ± 0.26 | 0.31 ± 0.28 | 13.48 ± 0.35 | 0.06 ± 0.005 | 1.8 ± 0.08 |
| 30-05-2022 | 1.64 ± 0.31 | 1.05 ± 0.03 | 3.24 ± 0.1 | 0.828 ± 0.075 | 1.96 ± 0.14 | 2.67 ± 0.43 | 0.5 ± 0.46 | 3.25 ± 0.08 | 0.19 ± 0.026 | 2.11 ± 0.16 |
| 09-06-2022 | 8.01 ± 0.59 | 0.69 ± 0.04 | 2.54 ± 0.05 | 0.22 ± 0.035 | 1.34 ± 0.08 | 7.36 ± 0.34 | 0.31 ± 0.36 | 2.55 ± 0.1 | 0.06 ± 0.005 | 1.8 ± 0.08 |
| 29-06-2022 | 6.93 ± 0.83 | 0.64 ± 0.01 | 3.16 ± 0.05 | 0.271 ± 0.019 | 1.38 ± 0.06 | 8.53 ± 0.79 | 0.29 ± 0.85 | 2.95 ± 0.05 | 0.17 ± 0.01 | 1.29 ± 0.07 |
| 23-07-2022 | 7.42 ± 0.54 | 0.63 ± 0.02 | 3.28 ± 0.08 | 0.424 ± 0.042 | 1.18 ± 0.09 | 5.63 ± 0.73 | 0.22 ± 0.79 | 3.09 ± 0.09 | 0.23 ± 0.011 | 1.24 ± 0.13 |
| 19-08-2022 | 0.93 ± 0.87 | 0.69 ± 0.04 | 3.83 ± 0.18 | 0.507 ± 0.118 | 2.79 ± 0.51 | 2.48 ± 0.47 | 0.31 ± 0.51 | 3.52 ± 0.13 | 0.31 ± 0.042 | 1.47 ± 0.35 |
| 07-09-2022 | -0.59 ± 0.93 | 0.71 ± 0.09 | 3.74 ± 0.12 | 0.409 ± 0.075 | 1.38 ± 0.21 | 0.09 ± 1.38 | 0.28 ± 1.48 | 3.74 ± 0.11 | 0.2 ± 0.025 | 1.37 ± 0.17 |
|  |  |  |  |  |  |  |  |  |  |  |

**
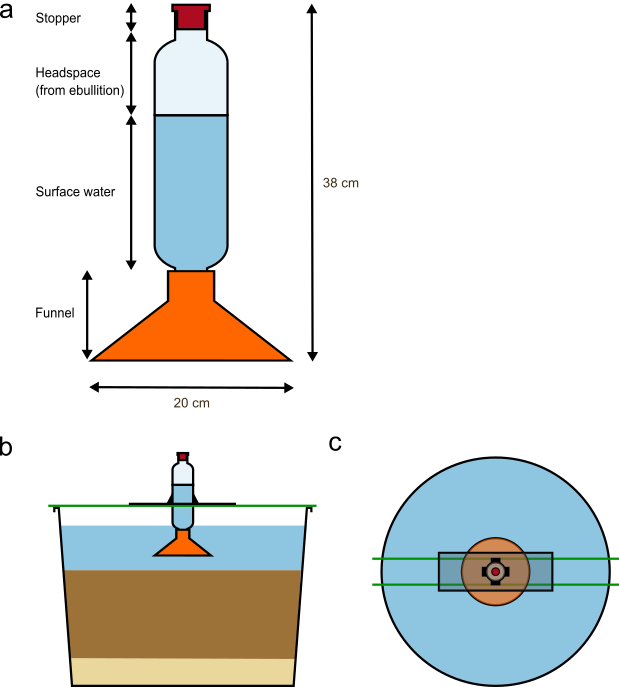
**

**Fig. S1** Schematic overview of the bubble trap construction, with a) side view, b) side view of installation within the mesocosm, and c) top view of installation within the mesocosm

**
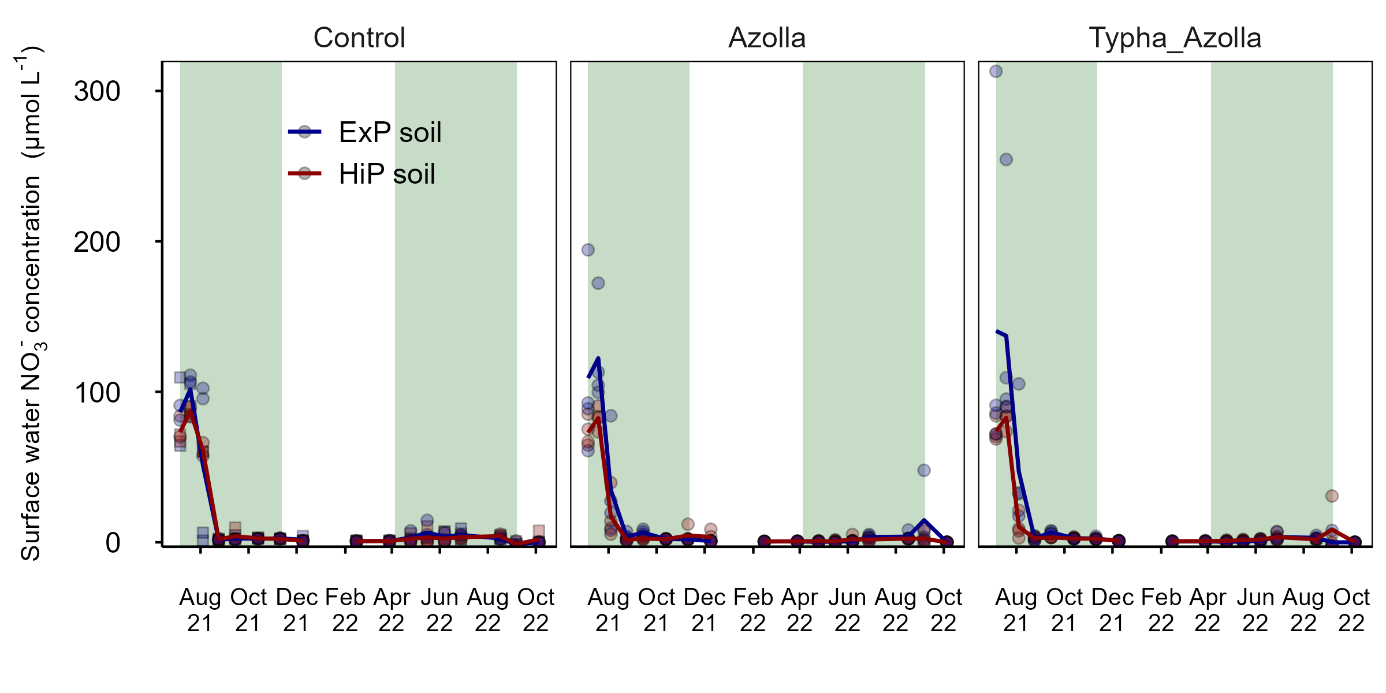
Fig. S2** Surface water NO_3_^-^ concentrations throughout the experiment. Lines represent average trends, symbols represent individual mesocosms. In controls, square symbols represent algae-dominated mesocosms while circles represent macrophyte-dominated mesocosms. Green shaded areas are periods of Azolla growth


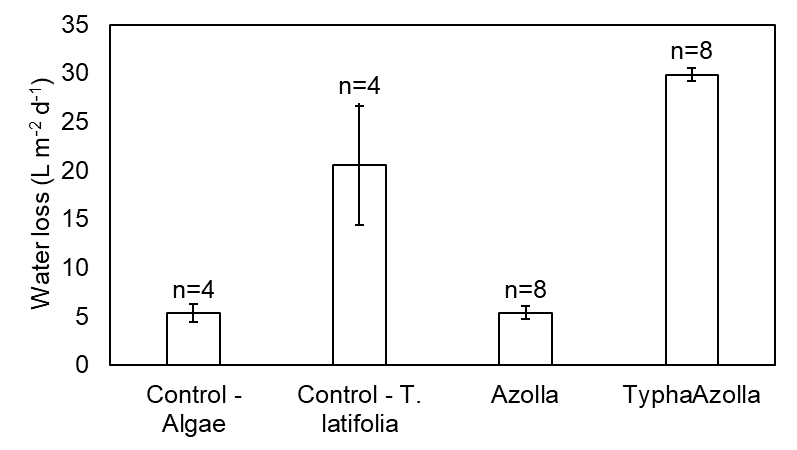


**Fig. S3** Water loss in the different vegetation treatments during a heat wave in summer 2022 (day temperatures >30 °C). Error bars represent standard errors


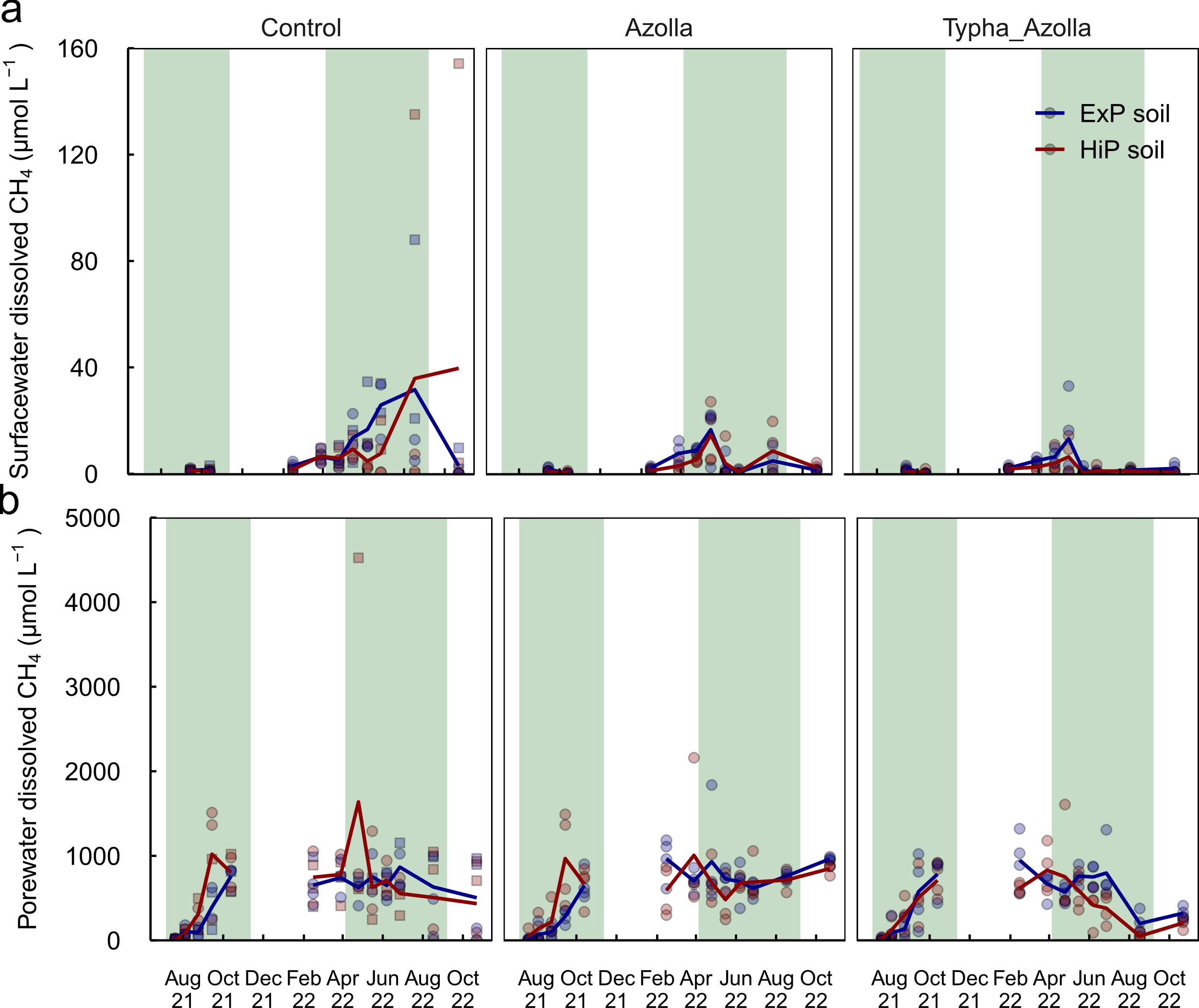


**Fig. S4** Dissolved CH_4_ in a) surface water and b) porewater over time. In controls, square symbols represent algae-dominated mesocosms while circles represent macrophyte-dominated mesocosms. Lines represent average trends, symbols represent individual mesocosms. Green shaded areas are periods in which *Azolla* was present.


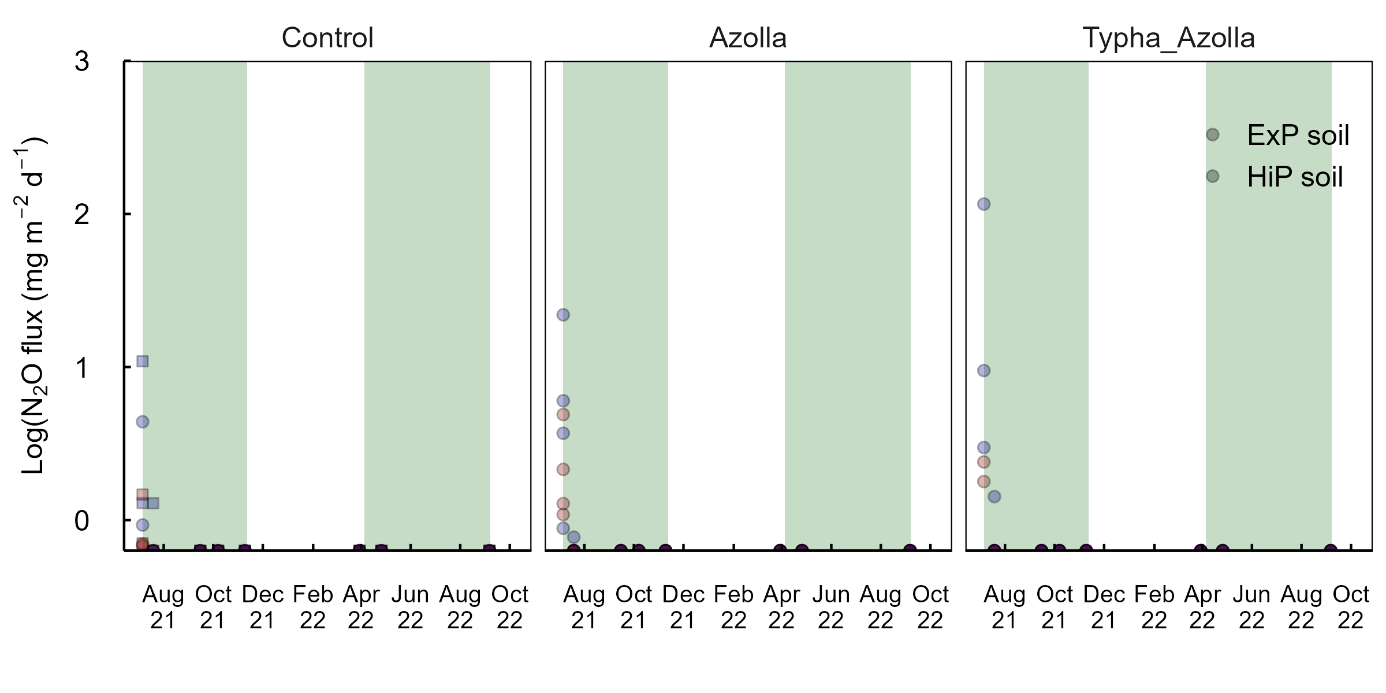


**Fig. S5** N_2_O emissions throughout the experiment (NB: logarithmic y-axis scale). Symbols represent individual mesocosms. In controls, square symbols represent algae-dominated mesocosms while circles represent macrophyte-dominated mesocosms. Green shaded areas are periods of Azolla growth.
